# Mental Health Promotion Among Black, Caribbean, and African Immigrants in Canada: Protocol for a Scoping Review

**DOI:** 10.2196/88586

**Published:** 2026-09-04

**Authors:** Noah Boakye-Yiadom, Rita Yembilah, Bukola Salami

**Affiliations:** 1 Department of Community Health Sciences, Cumming School of Medicine, University of Calgary, 2500 University Drive, Calgary, AB, T2N 1N4, Canada, 1 647 868 6624; 2 Department of Archeology and Anthropology, University of Calgary, Calgary, AB, Canada

**Keywords:** mental health promotion, Black, Caribbean, African, immigrant health, health equity

## Abstract

**Background:**

Black, Caribbean, and African immigrant communities in Canada face compounding systemic inequities that undermine mental well-being and limit access to culturally grounded mental health promotion (MHP) strategies. Despite growing national policy attention to these disparities, the landscape of MHP programs and initiatives developed for, with, or by Black, Caribbean, and African populations remains fragmented and poorly synthesized.

**Objective:**

This scoping review aims to (1) map existing MHP strategies, programs, and activities targeting Black, Caribbean, and African immigrant communities in Canada; (2) identify barriers and facilitators influencing their implementation and uptake; and (3) illuminate gaps in current research, policy, and practice.

**Methods:**

Guided by Arksey and O’Malley’s scoping review framework and the Joanna Briggs Institute Manual for Evidence Synthesis, the review draws on 6 databases: MEDLINE (OVID), APA PsycINFO (OVID), Embase (OVID), PubMed, CINAHL Plus (EBSCOhost), and Google Scholar. Gray literature sources include community reports and government-funded initiatives such as those supported by the Public Health Agency of Canada’s Promoting Health Equity: Mental Health of Black Canadians Fund. Eligibility criteria focus on English-language sources addressing MHP or mental illness prevention within the Canadian Black, Caribbean, and African immigrant context. Data will be charted independently in duplicate using Covidence and analyzed through descriptive methods, including tabular summaries and narrative synthesis.

**Results:**

Preliminary searches were conducted in June 2025. Formal database searches were completed in October 2025, yielding 2349 records across 6 databases. Following automated and manual deduplication (n=1048), 1301 records proceeded to title and abstract screening. Of these, 193 studies were sought for retrieval and full-text assessment. Data extraction is underway, with full results expected for publication in late 2026.

**Conclusions:**

This review will produce the first consolidated map of MHP strategies targeting Black, Caribbean, and African immigrant communities in Canada. Findings are expected to reveal heterogeneity in intervention design, with a concentration of reactive, treatment-adjacent programs and limited representation of upstream, community-led, and culturally rooted approaches. Results will inform equity-driven policy development, support culturally responsive service design, and provide a foundation for future participatory research that centers Black, Caribbean, and African communities as knowledge producers.

## Introduction

### Background

Mental health promotion (MHP) is a critical but often underresourced component of public health systems, particularly for racialized immigrant communities navigating layered social and structural inequities. Within the Canadian context, Black, Caribbean, and African immigrant populations face unique challenges to achieving mental well-being, including the compounded impacts of racial discrimination, migration stressors, intergenerational trauma, cultural dissonance, and barriers to accessing culturally safe care [1,2]. These experiences are intensified by the persistent erasure or tokenization of Black knowledge systems in mental health promotion design and delivery.

The concept of mental health promotion is defined as the process of enhancing protective factors and improving the social determinants that support psychological well-being, which requires contextual adaptation [3]. For Black, Caribbean, and African immigrants, MHP must move beyond biomedical models to encompass relational, cultural, and community-based understandings of wellness. Yet, despite national recognition of mental health inequities [4,5], efforts to systematically map and assess MHP interventions tailored to Black, Caribbean, and African immigrant communities remain limited.

Furthermore, the linguistic, cultural, generational, and migratory diversity within Black, Caribbean, and African populations necessitates mental health promotion efforts that are both inclusive and sensitive to variations within the community [6-9]. Current policy documents and funding streams (eg, Public Health Agency of Canada’s [PHAC’s] Promoting Health Equity: Mental Health of Black Canadians Fund) highlight a growing awareness of these needs, yet there is insufficient synthesis of what has been implemented, evaluated, or sustained over time.

This knowledge gap limits our ability to (1) understand which interventions are grounded in community realities, (2) learn from grassroots innovations or co-designed programs, (3) identify persistent barriers and underserved subgroups within the Black, Caribbean, and African immigrant population, and (4) inform future investment and capacity-building across systems of care.

A scoping review is therefore timely and necessary to consolidate fragmented knowledge, interrogate the equity implications of existing MHP strategies, and identify conceptual, methodological, and practice-based gaps.

### Study Rationale

#### Overview

This review is motivated by a critical imperative: to make visible the MHP efforts, formal and informal, that affect the lives of Black, Caribbean, and African immigrants in Canada. It is grounded in the recognition that these populations often navigate health systems that are not designed with their histories, values, or aspirations in mind. While research on mental health disparities in Black communities is growing, there is a lack of synthesis on proactive, upstream interventions that promote resilience, build community capacity, and affirm cultural identity.

The following 3 intersecting rationales guide this review.

#### Evidence Mapping

There is no consolidated map of MHP strategies or programs designed by, for, or with Black, Caribbean, and African immigrants in Canada. Existing interventions remain siloed across academic, policy, and community domains. A scoping review allows for a comprehensive synthesis across diverse sources and disciplines, including gray literature often excluded from traditional reviews.

#### Equity-Driven Systems Change

To achieve mental health equity, we must understand not only what works but also why, for whom, and under what conditions. By identifying barriers and facilitators within implementation contexts, such as trust in institutions, linguistic access, funding models, or diasporic identity dynamics, this review can generate actionable insights for more culturally responsive and structurally competent interventions.

#### Community-Affirming Research

This review aligns with calls for research approaches that reflect the lived realities and epistemologies of Black communities. In line with Africentric and decolonial paradigms, it seeks to shift the research gaze from deficit-focused accounts of mental illness to an asset-based understanding of mental health and community strength.

Ultimately, this review intends not only to inform practice and policy but also to serve as a foundation for future participatory and emancipatory research that centers Black, Caribbean, and African immigrant communities as knowledge producers in their own right.

### Review Objectives

This scoping review aims to comprehensively examine MHP initiatives targeting Black, Caribbean, and African immigrant communities in Canada. By mapping existing strategies, programs, and activities, this review seeks to provide a clear overview of the current efforts to support mental well-being within these populations. This mapping process will help identify successful approaches, innovative interventions, and areas where resources are being allocated effectively.

Furthermore, the review will examine the barriers and facilitators affecting the implementation and success of MHP initiatives in these communities. This analysis will shed light on cultural, social, economic, and systemic factors that may hinder or support MHP efforts. By identifying these barriers and facilitators, this review aims to inform future policy decisions, resource allocation, and program development to better address the unique mental health needs of Black, Caribbean, and African immigrants in Canada. Additionally, the review will highlight gaps in current research and practice, pointing toward areas that require further investigation or intervention to improve mental health outcomes for these populations.

Succinctly, the 3 main objectives of this scoping review are as follows:

1. Map the MHP strategies, programs, and activities targeting the Black, Caribbean, and African immigrant community in Canada.
2. Identify barriers and facilitators for MHP strategies, programs, and activities in the Black, Caribbean, and African immigrant community in Canada.
3. Identify gaps in current research on MHP for Black, Caribbean, and African immigrant communities in Canada.

## Methods

### Formative Work: Development of the Search Strategy

The search strategy was developed iteratively, following multiple preliminary searches and subsequent refinements. Initial searches were conducted using PubMed and Google Scholar databases. No temporal restrictions were imposed on published research.

Preliminary searches were conducted in June 2025. Only titles and abstracts were searched and extracted to data management tool Zotero (version 9.0.5; Digital Scholar). Formal, comprehensive searches across all 6 databases were completed in October 2025. The full search strings applied in each database are provided in Multimedia Appendix 1.

### Eligibility Criteria

The eligibility criteria for this study were developed from insights gained during preliminary searches conducted in June 2025 and were refined iteratively prior to the formal searches completed in October 2025. These criteria aim to focus the research on a specific population and context, ensuring that selected sources provide relevant and valuable information for the study’s objectives, as follows:

1. The paper is published in English
2. The population of focus is Black, Caribbean, and African immigrants
3. The setting is Canada
4. Includes MHP and mental illness prevention strategies, programs, and activities

The first criterion specifies that papers must be published in English, which facilitates a comprehensive review by the research team and ensures consistency in language interpretation. The population of focus is narrowed down to Black, Caribbean, and African immigrants, allowing for an in-depth exploration of mental health issues specific to these communities. By setting Canada as the geographical context, the study aims to examine the unique challenges and opportunities within the Canadian health care system and social environment. Finally, the focus on MHP and mental illness prevention highlights the study’s emphasis on proactive approaches to mental well-being rather than solely on treatment-oriented research. This comprehensive set of criteria will guide the selection of papers that contribute meaningfully to understanding and addressing the mental health needs of Black, Caribbean, and African immigrants in Canada.

### Information Sources

#### Literature Search Strategy

The literature search strategy encompasses 6 databases to ensure a comprehensive and diverse collection of relevant research. MEDLINE, accessed through the OVID platform, is a premier biomedical database providing extensive coverage of medical and health-related literature. APA PsycINFO, also accessed via OVID, focuses on psychological and behavioral sciences, offering insights into mental health and related fields. Embase, also accessed through OVID, covers a wide range of medical topics including health policy, mental health, and alternative medicine. PubMed, maintained by the National Center for Biotechnology Information, provides access to a broad range of biomedical and life sciences literature. CINAHL Plus (EBSCOhost) specializes in nursing and allied health research. Google Scholar, a widely used academic search engine, offers broad coverage of scholarly literature across disciplines, including articles, theses, books, and conference papers, and is particularly useful for identifying interdisciplinary research and gray literature. Together, these 6 databases ensure a thorough and well-rounded literature search capturing relevant studies from multiple perspectives and disciplines.

#### Methodological Framework

This review is guided by Arksey and O’Malley’s foundational scoping review framework [10], which provides a structured 5-stage process for mapping evidence across a broad topic area. To ensure methodological currency and rigor, the review is further informed by the Joanna Briggs Institute (JBI) Manual for Evidence Synthesis [11], which offers updated guidance on scoping review conduct, including source selection, data charting, and synthesis. Reporting will adhere to the PRISMA-ScR (Preferred Reporting Items for Systematic Reviews and Meta-Analyses extension for Scoping Reviews) guidelines [12]. A completed PRISMA-ScR checklist is provided as Checklist 1.

#### Grey Literature

Gray literature sources for this review include community reports, organizational publications, and government-funded initiatives relevant to MHP among Black, Caribbean, and African communities in Canada. One significant source is the Promoting Health Equity: Mental Health of Black Canadians Fund, established by the PHAC in 2018, which represents a significant step toward addressing the unique mental health challenges faced by Black Canadians. This initiative recognizes the intersectionality of race, mental health, and social determinants of health, acknowledging that Black Canadians often experience disproportionate barriers to accessing mental health services and support. The fund aims to support community-based projects that promote mental health awareness, reduce stigma, and improve access to culturally appropriate mental health resources for Black communities across Canada. Initiatives funded through this mechanism represent one category of gray literature to be examined; additional gray literature sources will be identified through targeted organizational searches and citation tracking as outlined in the search strategy.

### Search Strategy and Terms

The search strategy was structured around 3 core concepts, combined using the Boolean operator AND to connect each concept group. Within each concept group, synonymous and related terms were combined using the Boolean operator OR. The full, finalized search strings as applied in each database are provided in Multimedia Appendix 1. The 3 concept groups and their associated search terms are as follows:

1. Population: “Black” OR “Blacks” OR “Black people” OR “Caribbean” OR “Caribbeans” OR “Caribbean descent” OR “African” OR “African descent” OR “African immigrant”
2. Setting: “Canada” OR “Canadians” OR “in Canada”
3. Focus: “mental health promotion” OR “mental illness prevention” OR “mental health promotion strategy” OR “mental health promotion program” OR “mental health awareness” OR “stigma reduction” OR “mental health education” OR “mental health activities”

A complete search string therefore takes the form: [population terms] AND [setting terms] AND [focus terms], with database-specific syntax adjustments applied as documented in Multimedia Appendix 1. Where applicable, MeSH headings and controlled vocabulary terms were incorporated to enhance sensitivity. Searches were not restricted by date of publication.

### Data Charting

Data charting will be conducted using Covidence. Searches will be exported from each database into the systematic review online platform. Covidence performs automatic deduplication; 2 reviewers will independently screen titles and abstracts, resolving conflicts by consensus within the platform. The same reviewers will screen full texts, applying inclusion/exclusion criteria, and extract study characteristics and outcomes using Covidence’s structured forms. A third reviewer will verify all extractions for accuracy.

Conflicts between the 2 independent reviewers at any stage of screening or extraction will be resolved through discussion and consensus. Where consensus cannot be reached through discussion, the third reviewer will serve as an arbiter, and a final decision will be made by majority agreement.

The data-charting form is designed to capture the following information from each included source: study characteristics (author, year, country, study design, and methodology); participant characteristics (age, gender, health status, socioeconomic status, and racial or ethnic background); researcher characteristics (nationalities and fields of expertise); and study objectives and contextual factors. In addition, and centrally relevant to the review objectives, the form will capture characteristics of the MHP strategies themselves, including type of intervention or program; delivery modality (individual, group, community-level, or digital); duration and intensity; theoretical or conceptual framework used; target subgroup within the Black, Caribbean, and African population; reported outcomes or intended outcomes; and barriers and facilitators to implementation and uptake identified by study authors.

### Data Synthesis

The analysis and summarization of data will be conducted using descriptive methods, providing a comprehensive overview of the collected information. Tables and graphs will be used strategically to present study characteristics in a clear, visually appealing manner. These visual representations will be complemented by a narrative summary in the text, offering detailed explanations and interpretations of the data. This approach ensures that readers can easily grasp the key findings and trends identified in the study.

A comparative analysis of study characteristics and participant attributes will be conducted to identify research gaps. This process involves systematically examining similarities and differences across studies, considering factors such as methodologies, demographics, and outcomes. By conducting this thorough comparison, researchers can pinpoint areas where existing literature is lacking or inconsistent, highlighting opportunities for future investigations.

This approach not only contributes to a deeper understanding of the current state of research but also guides subsequent studies, ensuring they address relevant and unexplored aspects of the topic at hand.

### Ethical Considerations

This research does not involve human subjects or unpublished secondary data, eliminating the need for approval from an ethics committee focused on human research.

### Dissemination Plan

The results of the scoping review will be shared through various channels to reach both academic and community audiences. A peer-reviewed publication in a relevant public health or health equity journal is the primary vehicle. Results will also be presented at national and international conferences in community health sciences, public health, and health equity. Community-facing summaries will be developed in accessible formats for sharing with Black, Caribbean, and African community organizations, settlement agencies, and policymakers in Canada. Where appropriate, findings will be integrated into curriculum and training materials for health professionals working with Black, Caribbean, and African immigrant populations.

## Results

The search strategy was developed iteratively between May and June 2025, with preliminary searches conducted across PubMed and Google Scholar in June 2025. Formal, comprehensive searches across all 6 databases (MEDLINE, APA PsycINFO, Embase, PubMed, CINAHL Plus, and Google Scholar) were completed in October 2025. The full search strings applied in each database are provided in Multimedia Appendix 1.

**Figure 1.**
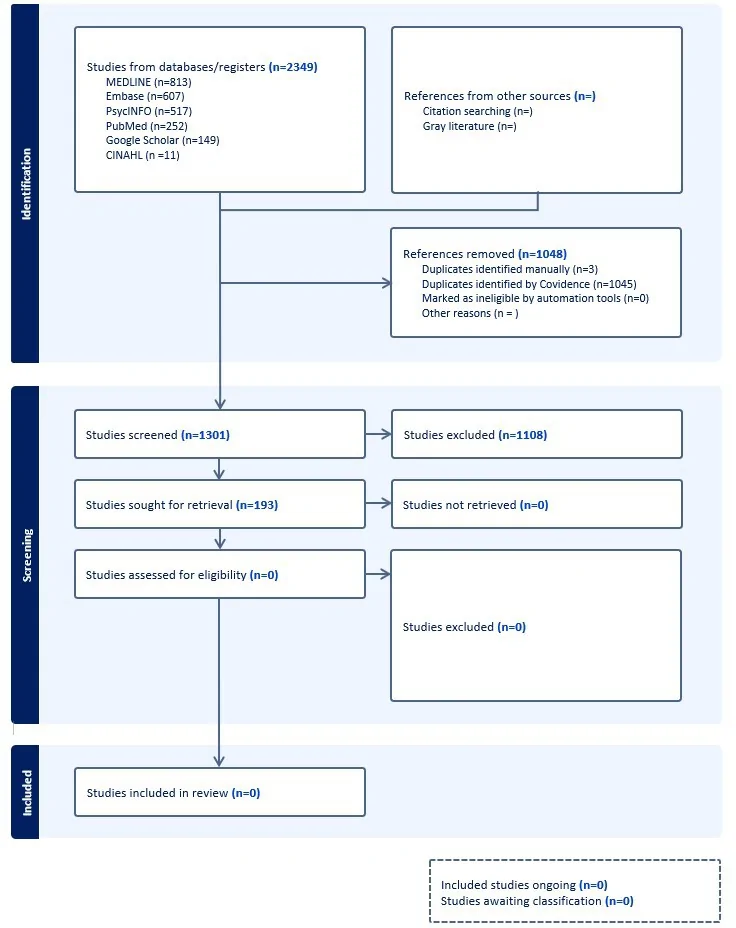
PRISMA (Preferred Reporting Items for Systematic Reviews and Meta-Analyses) flow diagram.

The database searches yielded a combined total of 2349 records from MEDLINE (n=813), Embase (n=607), APA PsycINFO (n=517), PubMed (n=252), Google Scholar (n=149), and CINAHL Plus (n=11). Following automated deduplication in Covidence (n=1045) and manual deduplication (n=3), a total of 1048 duplicate records were removed, yielding 1301 unique records for title and abstract screening. Of these, 193 studies were identified as potentially eligible and sought for full-text retrieval and assessment. Gray literature searching, citation searching, and full-text eligibility assessment are in progress as part of the ongoing data extraction phase. Final counts for records retrieved, assessed for eligibility, excluded at full-text review, and included in the review will be reported in the full review publication, expected in late 2026. A PRISMA (Preferred Reporting Items for Systematic Reviews and Meta-Analyses) flow diagram illustrating the search and screening process is provided as Figure 1.

## Discussion

### Principal Findings

This scoping review is designed to produce the first consolidated synthesis of MHP strategies, programs, and activities targeting Black, Caribbean, and African immigrant communities in Canada. Based on the objectives and the landscape of literature identified through preliminary and formal searches, several anticipated principal findings can be articulated. First, existing MHP efforts are expected to reflect considerable heterogeneity in design, scope, and theoretical grounding, with variation across program type (individual, family-based, and community-level), delivery modality (clinical, community, and digital), and population subgroup. Second, the review anticipates that upstream, preventive, and community-governed MHP approaches will be underrepresented relative to reactive, treatment-adjacent, or clinically framed interventions. Third, the evidence base is expected to reveal persistent gaps in the representation of grassroots, diasporic, and Africentric frameworks within formal research and policy documentation, reflecting the broader structural marginalization of Black knowledge systems in Canadian public health. These anticipated findings will be examined and refined as data extraction is completed.

### Comparison to Prior Work

Existing reviews on the mental health of Black populations in Canada have primarily focused on mental health service utilization, barriers to care, and the epidemiology of mental disorders rather than on proactive, upstream MHP strategies [2,4]. A 2024 scoping review by Martínez-Vega and colleagues examined mental health standards for Black youth across community, primary care, and educational settings, identifying substantial equity gaps; however, the review did not systematically address MHP programming for Black, Caribbean, and African immigrant adults or examine the role of community-led or culturally rooted interventions. Similarly, work by Alaazi and colleagues [1] examined community- and family-based approaches to child MHP among African immigrants in Alberta, offering important community-level insights that nonetheless remain geographically and demographically bounded. This review will build on and extend these contributions by focusing explicitly on the immigrant experience across Black, Caribbean, and African subgroups, incorporating gray literature and community-based sources, and centering equity-driven and decolonial analytical lenses.

### Strengths and Limitations

This review carries several methodological strengths. The use of 6 databases alongside gray literature sources broadens the evidence base beyond peer-reviewed academic literature, capturing community-led innovations and policy-driven initiatives that are frequently excluded from narrower reviews. The application of both Arksey and O’Malley’s scoping review framework and JBI guidance ensures methodological rigor and transparency. Independent duplicate data charting with a third-reviewer verification process strengthens interrater reliability.

Nonetheless, several limitations warrant acknowledgment. The restriction to English-language sources may exclude relevant francophone literature, particularly given the significant Black, Caribbean, and African immigrant population in Quebec and other francophone regions. The heterogeneity of the Black, Caribbean, and African population, spanning diverse national origins, migration histories, generational statuses, and linguistic communities, means that aggregate findings may obscure important within-group variation. Additionally, the reliance on formal publication and indexed gray literature may underrepresent informal, oral, or community-held knowledge that does not circulate in documented form. These limitations do not undermine the review’s contributions but should be considered when interpreting and applying its findings.

### Future Directions

The findings of this review are intended to serve as a foundation for subsequent research phases. In particular, gaps identified through the mapping process will inform the design of participatory and community-based interventions that center Black, Caribbean, and African immigrants as coinvestigators and knowledge producers. Future work may also examine the implementation fidelity and long-term sustainability of identified MHP programs, areas that scoping reviews are not designed to assess but that are critical for translating evidence into sustained practice and policy change.

## Supplementary material

10.2196/88586 Multimedia Appendix 1 Detailed search strategies.

10.2196/88586 Checklist 1 PRISMA-ScR Checklist.

## Abbreviations

JBI: Joanna Briggs Institute
MHP: mental health promotion
PHAC: Public Health Agency of Canada
PRISMA: Preferred Reporting Items for Systematic Reviews and Meta-Analyses
PRISMA-ScR: Preferred Reporting Items for Systematic Reviews and Meta-Analyses extension for Scoping Reviews
