# Supplementary material for: Mental Health Promotion Among Black, Caribbean, and African Immigrants in Canada: Protocol for a Scoping Review
Source: JMIR Res Protoc. 2026 Sep 4;15:e88586. doi: 10.2196/88586 (PMC13544689; doi:10.2196/88586)
Supplement: Supplementary file 1 [file resprot-v15-e88586-s001.docx]

## **Appendix**

**The following strings were run between July 8, 2025 – July 21, 2025**

**OVID String search**

1. exp Blacks/ or Afro Caribbeans/ or Immigrants/
2. black*.ti,ab,kw. or african*.ti,ab,kw. or caribbean*.ti,ab,kw.
3. 1 or 2
4. Canada/
5. canada*.ti,ab,kw. or "in Canada".ti,ab,kw.
6. 4 or 5
7. exp Mental Health Programs/ or Mental Illness Prevention/ or exp Health Promotion/
8. ("mental health promotion" or "mental illness prevention" or "stigma reduction" or "mental health education" or "mental health activit*").ti,ab,kw.
9. 7 or 8
10. 3 and 6 and 9
11. limit 10 to English language

run with with all provinces and major cities as settings

include all African countries and Caribbean country names in population searches.

**PUBMED Search String**

| **Concept** | **MeSH / Supplementary** | **Free‑text (field tag [tiab])** |
| --- | --- | --- |
| Population | "African Continental Ancestry Group"[Mesh] OR "Caribbean Region"[Mesh] | black*[tiab] OR african*[tiab] OR caribbean*[tiab] OR "african immigrant*"[tiab] |
| Setting | "Canada"[Mesh] | canada*[tiab] OR "in Canada"[tiab] |
| Focus | "Mental Health"[Mesh] OR "Health Promotion"[Mesh] OR "Mental Disorders/prevention and control"[Mesh] | "mental health promotion"[tiab] OR "mental illness prevention"[tiab] OR "stigma reduction"[tiab] OR "mental health education"[tiab] OR "mental health activit*"[tiab] |

**Google Scholar Search String**

- ("mental health promotion" OR "mental illness prevention" OR "stigma reduction" OR "mental health education")
- AND
- (Black OR African OR Caribbean)
- AND
- Canada

**CINAHL Plus (EBSCOhost)**

| Concept | CINAHL Headings | Free-text (TI,AB) |
| --- | --- | --- |
| Population | MH "Blacks+" OR MH "African Continental Ancestry Group+" OR MH "African Diaspora" | black* OR african* OR caribbean* |
| Setting | MH "Canada" | canada* OR "in canada" |
| Focus | MH "Mental Health Promotion" OR MH "Primary Prevention" OR MH "Health Promotion" | "mental health promotion" OR "mental illness prevention" OR "stigma reduction" OR "mental health education" OR "mental health activit*" |

**The following strings were run from July 30 – August 5, 2025**

*The following strategy was used to expand the setting to capture settings in Canadian provinces and major cities that did not explicitly mention Canada.*

**PUBMED**

| **Concept** | **MeSH / Supplementary** | **Free‑text (field tag [tiab])** |
| --- | --- | --- |
| Population | "African Continental Ancestry Group"[Mesh] OR "Caribbean Region"[Mesh] | black*[tiab] OR african*[tiab] OR caribbean*[tiab] OR "african immigrant*"[tiab] |
| Setting | "Canada"[Mesh] OR  "Alberta"[Mesh] OR "British Columbia"[Mesh] OR "Manitoba"[Mesh] OR  "New Brunswick"[Mesh] OR "Newfoundland and Labrador"[Mesh] OR  "Nova Scotia"[Mesh] OR "Ontario"[Mesh] OR "Prince Edward Island"[Mesh] OR  "Quebec"[Mesh] OR "Saskatchewan"[Mesh] | canada*[tiab] OR "in Canada"[tiab] canada*[tiab] OR alberta[tiab] OR "british columbia"[tiab] OR manitoba[tiab] OR  "new brunswick"[tiab] OR "newfoundland and labrador"[tiab] OR  "nova scotia"[tiab] OR ontario[tiab] OR "prince edward island"[tiab] OR  quebec[tiab] OR saskatchewan[tiab] OR toronto[tiab] OR montréal[tiab] OR montreal[tiab] OR ottawa[tiab] OR  edmonton[tiab] OR calgary[tiab] OR winnipeg[tiab] OR  vancouver[tiab] OR hamilton[tiab] OR oshawa[tiab] OR  "quebec city"[tiab] OR "québec city"[tiab] |
| Focus | "Mental Health"[Mesh] OR "Health Promotion"[Mesh] OR "Mental Disorders/prevention and control"[Mesh] | "mental health promotion"[tiab] OR "mental illness prevention"[tiab] OR "stigma reduction"[tiab] OR "mental health education"[tiab] OR "mental health activit*"[tiab] |

**OVID Search String**

1. exp Blacks/ or Afro Caribbeans/ or Immigrants/
2. black*.ti,ab,kw. or african*.ti,ab,kw. or caribbean*.ti,ab,kw.
3. 1 or 2
4. Canada/ or Ontario/ or Quebec/ or Alberta/ or "British Columbia"/ or

Saskatchewan/ or Manitoba/ or "Prince Edward Island"/ or

"Nova Scotia"/ or "New Brunswick"/ or "Newfoundland and Labrador"/

5. (canada* or "in canada" or alberta or "british columbia" or saskatchewan or manitoba or ontario or "prince edward island" or "new brunswick" or "nova scotia" or "newfoundland and labrador" or toronto or montreal or ottawa or hamilton or halifax or edmonton or calgary or winnipeg or regina or vancouver or "quebec city" or victoria).ti,ab,kw.
6. 4 or 5
7. exp Mental Health Programs/ or Mental Illness Prevention/ or exp Health Promotion/ or mental health/
8. (“mental health” or "mental health promotion" or "mental illness prevention" or "stigma reduction" or "mental health education" or "mental health activit*").ti,ab,kw.
9. 7 or 8
10. 3 and 6 and 9
11. limit 10 to English language

*The following was used to increase the population search.*

**Searches from July 31 to August 10, 2025**

## ***Full country list used (for transparency)***

Sub-Saharan Africa (46 UN-defined states):
Angola, Benin, Botswana, Burkina Faso, Burundi, Cabo Verde, Cameroon, Central African Republic, Chad, Comoros, Congo, Democratic Republic of Congo, Côte d’Ivoire, Djibouti, Equatorial Guinea, Eritrea, Eswatini, Ethiopia, Gabon, Gambia, Ghana, Guinea, Guinea-Bissau, Kenya, Lesotho, Liberia, Madagascar, Malawi, Mali, Mauritania, Mauritius, Mozambique, Namibia, Niger, Nigeria, Rwanda, São Tomé and Príncipe, Senegal, Seychelles, Sierra Leone, Somalia, South Africa, South Sudan, Sudan, Tanzania, Togo, Uganda, Zambia, Zimbabwe.

Caribbean sovereign states & principal territories (22):
Antigua & Barbuda, Bahamas, Barbados, Belize, Cuba, Dominica, Dominican Republic, Grenada, Guyana, Haiti, Jamaica, Saint Kitts & Nevis, Saint Lucia, Saint Vincent & the Grenadines, Suriname, Trinidad & Tobago, plus Aruba, Curaçao, Guadeloupe, Martinique, Puerto Rico, US/British Virgin Islands.

**PUBMED**

| **Concept** | **MeSH / Supplementary** | **Free‑text (field tag [tiab])** |
| --- | --- | --- |
| Population | "African Continental Ancestry Group"[Mesh] OR "Caribbean Region"[Mesh] | black*[tiab] OR african*[tiab] OR caribbean*[tiab] OR "african immigrant*"[tiab]  /* sub-Saharan countries (root* to pick up demonyms) */  OR angola*[tiab] OR benin*[tiab] OR botswana*[tiab] OR "burkina faso"[tiab] OR burkinab*[tiab]  OR burundi*[tiab] OR "cabo verde"[tiab] OR "cape verde"[tiab] OR cameroon*[tiab]  OR "central african"[tiab] OR chad*[tiab] OR comoro*[tiab] OR congo*[tiab]  OR "democratic republic of congo"[tiab] OR zair*[tiab] OR "cote d’ivoire"[tiab] OR ivoir*[tiab]  OR djibouti*[tiab] OR "equatorial guinea"[tiab] OR equatoguinean*[tiab]  OR eritrea*[tiab] OR eswatini*[tiab] OR swazi*[tiab] OR ethiopia*[tiab]  OR gabon*[tiab] OR gambia*[tiab] OR gambian*[tiab] OR ghana*[tiab]  OR guinea[tiab] OR "guinea-bissau"[tiab] OR kenyan*[tiab] OR kenya[tiab]  OR lesotho*[tiab] OR liberia*[tiab] OR madagascar*[tiab] OR malawi*[tiab]  OR mali*[tiab] OR mauritania*[tiab] OR mauritius*[tiab] OR mozambi*[tiab]  OR namibia*[tiab] OR niger[^a-z][tiab] OR nigerien*[tiab] OR nigeria*[tiab]  OR rwanda*[tiab] OR "sao tome"[tiab] OR principe*[tiab] OR senegal*[tiab]  OR seychell*[tiab] OR "sierra leone"[tiab] OR somali*[tiab]  OR "south africa"[tiab] OR "south african"[tiab] OR "south sudan"[tiab]  OR sudan*[tiab] OR tanzan*[tiab] OR togo*[tiab] OR uganda*[tiab]  OR zambi*[tiab] OR zimbabwe*[tiab]  /* Caribbean & diaspora */  OR antigua*[tiab] OR bahama*[tiab] OR barbados*[tiab] OR belize*[tiab]  OR cuba*[tiab] OR dominica[tiab] OR "dominican republic"[tiab]  OR grenada*[tiab] OR guyan*[tiab] OR haiti*[tiab] OR haitian*[tiab]  OR jamaica*[tiab] OR kittitian*[tiab] OR nevis*[tiab] OR "saint kitts"[tiab]  OR lucian*[tiab] OR "saint lucia"[tiab]  OR "saint vincent"[tiab] OR vincentian*[tiab] OR grenadine*[tiab]  OR surinam*[tiab] OR trinidad*[tiab] OR tobago*[tiab]  OR aruba*[tiab] OR curaçao*[tiab] OR curacao*[tiab]  OR guadeloupe*[tiab] OR martinique*[tiab]  OR "puerto rico"[tiab] OR boricua*[tiab] OR "virgin islands"[tiab] |
| Setting | "Canada"[Mesh] OR  "Alberta"[Mesh] OR "British Columbia"[Mesh] OR "Manitoba"[Mesh] OR  "New Brunswick"[Mesh] OR "Newfoundland and Labrador"[Mesh] OR  "Nova Scotia"[Mesh] OR "Ontario"[Mesh] OR "Prince Edward Island"[Mesh] OR  "Quebec"[Mesh] OR "Saskatchewan"[Mesh] | canada*[tiab] OR "in Canada"[tiab] canada*[tiab] OR alberta[tiab] OR "british columbia"[tiab] OR manitoba[tiab] OR  "new brunswick"[tiab] OR "newfoundland and labrador"[tiab] OR  "nova scotia"[tiab] OR ontario[tiab] OR "prince edward island"[tiab] OR  quebec[tiab] OR saskatchewan[tiab] OR toronto[tiab] OR montréal[tiab] OR montreal[tiab] OR ottawa[tiab] OR  edmonton[tiab] OR calgary[tiab] OR winnipeg[tiab] OR  vancouver[tiab] OR hamilton[tiab] OR oshawa[tiab] OR  "quebec city"[tiab] OR "québec city"[tiab] |
| Focus | "Mental Health"[Mesh] OR "Health Promotion"[Mesh] OR "Mental Disorders/prevention and control"[Mesh] | "mental health promotion"[tiab] OR "mental illness prevention"[tiab] OR "stigma reduction"[tiab] OR "mental health education"[tiab] OR "mental health activit*"[tiab] |

**OVID Search String MEDLINE and EMBASE**

1. exp African Continental Ancestry Group/ or exp Caribbean Region/

2. (black* or afro-canadian* or african* or caribbean*

or angola* or benin* or botswana* or "burkina faso" or burkinab* or burundi*

or "cabo verde" or "cape verde" or cameroon* or "central african" or chad*

or comoro* or congo* or "democratic republic of congo" or zair*

or "cote d'ivoire" or ivoir* or djibouti* or "equatorial guinea" or equatoguinean*

or eritrea* or eswatini* or swazi* or ethiopia* or gabon* or gambia* or gambian*

or ghana* or guinea or "guinea-bissau" or kenyan* or kenya or lesotho* or liberia*

or madagascar* or malawi* or mali* or mauritania* or mauritius* or mozambi*

or namibia* or niger or nigerien* or nigeria* or rwanda* or "sao tome" or principe*

or senegal* or seychell* or "sierra leone" or somali* or "south africa"

or "south sudan" or sudan* or tanzan* or togo* or uganda* or zambi* or zimbabwe*

or antigua* or bahama* or barbados* or belize* or cuba* or dominica

or "dominican republic" or grenada* or guyan* or haiti* or haitian*

or jamaica* or "saint kitts" or kittitian* or nevis* or "saint lucia" or lucian*

or "saint vincent" or vincentian* or grenadine* or surinam* or trinidad* or tobago*

or aruba* or curacao* or guadeloupe* or martinique* or "puerto rico" or "virgin islands").ti,ab,kw.

3. 1 or 2

4. exp Canada/

5. (canada* or "in canada" or alberta or "british columbia" or saskatchewan or manitoba or

ontario or "prince edward island" or "new brunswick" or "nova scotia" or

"newfoundland and labrador" or toronto or montreal or ottawa or hamilton or halifax

or edmonton or calgary or winnipeg or regina or vancouver or "quebec city" or victoria).ti,ab,kw.

6. 4 or 5

7. exp Mental Health/ or exp Health Promotion/ or Mental Disorders/pc

8. ("mental health promotion" or "mental illness prevention" or

"mental health promotion strategy" or "mental health promotion program" or

"mental health awareness" or "stigma reduction" or

"mental health education" or "mental health activit*").ti,ab,kw.

9. 7 or 8

10. 3 and 6 and 9

**OVID Search String APA PsychINFO**

1. exp Blacks/ or exp Afro Caribbeans/ or exp Immigrants/

2. (black* or afro-canadian* or african* or caribbean*

or angola* or benin* or botswana* or "burkina faso" or burkinab* or burundi*

or "cabo verde" or "cape verde" or cameroon* or "central african" or chad*

or comoro* or congo* or "democratic republic of congo" or zair*

or "cote d'ivoire" or ivoir* or djibouti* or "equatorial guinea" or equatoguinean*

or eritrea* or eswatini* or swazi* or ethiopia* or gabon* or gambia* or gambian*

or ghana* or guinea or "guinea-bissau" or kenyan* or kenya or lesotho* or liberia*

or madagascar* or malawi* or mali* or mauritania* or mauritius* or mozambi*

or namibia* or niger or nigerien* or nigeria* or rwanda* or "sao tome" or principe*

or senegal* or seychell* or "sierra leone" or somali* or "south africa"

or "south sudan" or sudan* or tanzan* or togo* or uganda* or zambi* or zimbabwe*

or antigua* or bahama* or barbados* or belize* or cuba* or dominica

or "dominican republic" or grenada* or guyan* or haiti* or haitian*

or jamaica* or "saint kitts" or kittitian* or nevis* or "saint lucia" or lucian*

or "saint vincent" or vincentian* or grenadine* or surinam* or trinidad* or tobago*

or aruba* or curacao* or guadeloupe* or martinique* or "puerto rico" or "virgin islands").ti,ab,kw.

3. 1 or 2

4. exp Canada/

5. (canada* or "in canada" or alberta or "british columbia" or saskatchewan or manitoba or

ontario or "prince edward island" or "new brunswick" or "nova scotia" or

"newfoundland and labrador" or toronto or montreal or ottawa or hamilton or halifax

or edmonton or calgary or winnipeg or regina or vancouver or "quebec city" or victoria).ti,ab,kw.

6. 4 or 5

7. exp Mental Health Programs/ or Mental Illness Prevention/ or exp Health Promotion/

8. ("mental health promotion" or "mental illness prevention" or

"mental health promotion strategy" or "mental health promotion program" or

"mental health awareness" or "stigma reduction" or

"mental health education" or "mental health activit*").ti,ab,kw.

9. 7 or 8

10. 3 and 6 and 9

**EBSCOhost Search String for (CINAHL plus)**

(

(MH "Blacks+") OR (MH "African Continental Ancestry Group+") OR (MH "Caribbean Region+") OR

TX(black* OR afro-canadian* OR african* OR caribbean* OR

angola* OR benin* OR botswana* OR "burkina faso" OR burkinab* OR burundi* OR

"cabo verde" OR "cape verde" OR cameroon* OR "central african" OR chad* OR

comoro* OR congo* OR "democratic republic of congo" OR zair* OR

"cote d'ivoire" OR ivoir* OR djibouti* OR "equatorial guinea" OR equatoguinean* OR

eritrea* OR eswatini* OR swazi* OR ethiopia* OR gabon* OR gambia* OR gambian* OR

ghana* OR guinea OR "guinea-bissau" OR kenyan* OR kenya OR lesotho* OR liberia* OR

madagascar* OR malawi* OR mali* OR mauritania* OR mauritius* OR mozambi* OR

namibia* OR niger OR nigerien* OR nigeria* OR rwanda* OR "sao tome" OR principe* OR

senegal* OR seychell* OR "sierra leone" OR somali* OR "south africa" OR

"south sudan" OR sudan* OR tanzan* OR togo* OR uganda* OR zambi* OR zimbabwe* OR

antigua* OR bahama* OR barbados* OR belize* OR cuba* OR dominica OR

"dominican republic" OR grenada* OR guyan* OR haiti* OR haitian* OR jamaica* OR

"saint kitts" OR kittitian* OR nevis* OR "saint lucia" OR lucian* OR

"saint vincent" OR vincentian* OR grenadine* OR surinam* OR trinidad* OR tobago* OR

aruba* OR curacao* OR guadeloupe* OR martinique* OR "puerto rico" OR "virgin islands")

)

AND

(

(MH "Canada+") OR

TX(canada* OR "in canada" OR alberta OR "british columbia" OR saskatchewan OR manitoba OR

ontario OR "prince edward island" OR "new brunswick" OR "nova scotia" OR

"newfoundland and labrador" OR toronto OR montreal OR ottawa OR hamilton OR halifax OR

edmonton OR calgary OR winnipeg OR regina OR vancouver OR "quebec city" OR victoria)

)

AND

(

(MH "Mental Health Promotion") OR (MH "Health Promotion") OR (MH "Primary Prevention") OR

TX("mental health promotion" OR "mental illness prevention" OR

"mental health promotion strategy" OR "mental health promotion program" OR

"mental health awareness" OR "stigma reduction" OR

"mental health education" OR "mental health activit*")

)

**Google Scholar Search String** ~ 256 character limitations

("mental health promotion" OR "mental illness prevention" OR "mental health education")

(Black OR African OR Caribbean OR Ghanaian OR Nigerian OR Jamaican OR Haitian OR Somali OR Ethiopian)

(Canada OR Alberta OR Ontario OR Quebec OR "British Columbia" OR Toronto OR Montreal OR Ottawa OR Edmonton OR Calgary)

First search

("stigma reduction") (Black OR African OR Caribbean OR Ghanaian OR Nigerian OR Jamaican OR Haitian OR Somali OR Ethiopian)

(Canada OR Alberta OR Ontario OR Quebec OR "British Columbia" OR Toronto OR Montreal OR Ottawa OR Edmonton OR Calgary)

Second search

("mental health activities") (Black OR African OR Caribbean OR Ghanaian OR Nigerian OR Jamaican OR Haitian OR Somali OR Ethiopian)

(Canada OR Alberta OR Ontario OR Quebec OR "British Columbia" OR Toronto OR Montreal OR Ottawa OR Edmonton OR Calgary)
