# Supplementary material for: Mental Health Promotion Among Black, Caribbean, and African Immigrants in Canada: Protocol for a Scoping Review
Source: JMIR Res Protoc. 2026 Sep 4;15:e88586. doi: 10.2196/88586 (PMC13544689; doi:10.2196/88586)
Supplement: Supplementary file 2 [file resprot-v15-e88586-s002.docx]

**PRISMA Extension for Scoping Reviews (PRISMA-ScR) Checklist**

*Manuscript: Mental Health Promotion among Black, Caribbean and African Immigrants in Canada: A Scoping Review Protocol*

JMIR Research Protocols | Manuscript #88586

Completed against the revised manuscript (88586-1305609-1-RV.docx). Page numbers reflect rendered pages in the submitted document. Items 15-18 marked N/A as this is a protocol; data will be reported in the full review publication (expected late 2026).

| **SECTION** | **ITEM** | **PRISMA-ScR CHECKLIST ITEM** | **REPORTED ON PAGE #** | **NOTES** |
| --- | --- | --- | --- | --- |
| **TITLE** | | | | |
| Title | **1** | Identify the report as a scoping review. | **1** |  |
| **ABSTRACT** | | | | |
| Structured summary | **2** | Provide a structured summary that includes (as applicable): background, objectives, eligibility criteria, sources of evidence, charting methods, results, and conclusions that relate to the review questions and objectives. | **1–2** | *Abstract structured as Background, Objectives, Methods, Results, and Conclusions. All required elements addressed.* |
| **INTRODUCTION** | | | | |
| Rationale | **3** | Describe the rationale for the review in the context of what is already known. Explain why the review questions/objectives lend themselves to a scoping review approach. | **3–4** | *Background and Study Rationale sections address the knowledge gap and justify the scoping review approach.* |
| Objectives | **4** | Provide an explicit statement of the questions and objectives being addressed with reference to their key elements (e.g., population or participants, concepts, and context) or other relevant key elements used to conceptualize the review questions and/or objectives. | **4–5** | *Three objectives stated explicitly in Review Objectives section: mapping MHP strategies, identifying barriers/facilitators, and identifying research gaps.* |
| **METHODS** | | | | |
| Protocol and registration | **5** | Indicate whether a review protocol exists; state if and where it can be accessed (e.g., a Web address); and if available, provide registration information, including the registration number. | N/A | *This manuscript IS the protocol under review at JMIR Research Protocols. Registration number not applicable at this stage.* |
| Eligibility criteria | **6** | Specify characteristics of the sources of evidence used as eligibility criteria (e.g., years considered, language, and publication status), and provide a rationale. | **6** | *Eligibility criteria section: English-language; BCA immigrant population; Canada setting; MHP or mental illness prevention focus. Rationale provided for each criterion. No date restrictions applied.* |
| Information sources* | **7** | Describe all information sources in the search (e.g., databases with dates of coverage and contact with authors to identify additional sources), as well as the date the most recent search was executed. | **6–7** | *Six databases named with platforms: MEDLINE (OVID), APA PsycINFO (OVID), EMBASE (OVID), PubMed, CINAHL Plus (EBSCOhost), Google Scholar. Grey literature sources described. Formal searches completed October 2025.* |
| Search | **8** | Present the full electronic search strategy for at least one database, including any limits used, such that it could be repeated. | **7–8 & Multimedia Appendix 1** | *Search strategy and terms section presents the three concept groups and Boolean logic. Full finalized search strings for all six databases provided in Multimedia Appendix 1.* |
| Selection of sources of evidence† | **9** | State the process for selecting sources of evidence (i.e., screening and eligibility) included in the scoping review. | **8–9** | *Data charting section describes independent duplicate screening of titles/abstracts and full texts by two reviewers in Covidence, applying inclusion/exclusion criteria, with a third reviewer verifying extractions.* |
| Data charting process‡ | **10** | Describe the methods of charting data from the included sources of evidence (e.g., calibrated forms or forms that have been pilot-tested by the team before their use, and whether data charting was done independently or in duplicate) and any processes for obtaining and confirming data from investigators. | **8–9** | *Data charted independently in duplicate using Covidence structured forms. Third reviewer verifies all extractions. Conflict resolution procedure described (discussion then arbiter).* |
| Data items | **11** | List and define all variables for which data were sought and any assumptions made and simplifications used. | **8–9** | *Data charting section lists variables: study characteristics, participant characteristics, researcher characteristics, study objectives, and MHP strategy variables (type, modality, duration, framework, target subgroup, outcomes, barriers/facilitators).* |
| Critical appraisal of individual sources of evidence§ | **12** | If done, provide a rationale for conducting a critical appraisal of included sources of evidence; describe the methods used and how this information was used in any data synthesis. | N/A | *Critical appraisal not conducted. Consistent with scoping review methodology; risk of bias assessment is optional and not standard practice for scoping reviews.* |
| Synthesis of results | **13** | Describe the methods of handling and summarizing the data that were charted. | **9** | *Data synthesis section describes descriptive methods: tables, graphs, and narrative summary to identify thematic trends and research gaps. Comparative analysis across study characteristics described.* |
| **RESULTS** | | | | |
| Selection of sources of evidence | **14** | Give numbers of sources of evidence screened, assessed for eligibility, and included in the review, with reasons for exclusions at each stage, ideally using a flow diagram. | **10 & Figure 1** | *Results section reports: 2,349 records identified; 1,048 duplicates removed; 1,301 screened; 193 sought for full-text retrieval. PRISMA flow diagram provided as Figure 1. Final inclusion counts pending completion of data extraction; to be reported in the full review publication.* |
| Characteristics of sources of evidence | **15** | For each source of evidence, present characteristics for which data were charted and provide the citations. | N/A | *Protocol manuscript. Characteristics of included sources will be reported in the full scoping review publication, expected late 2026.* |
| Critical appraisal within sources of evidence | **16** | If done, present data on critical appraisal of included sources of evidence (see item 12). | N/A | *Critical appraisal not conducted; see item 12.* |
| Results of individual sources of evidence | **17** | For each included source of evidence, present the relevant data that were charted that relate to the review questions and objectives. | N/A | *Protocol manuscript. Individual source results will be reported in the full scoping review publication, expected late 2026.* |
| Synthesis of results | **18** | Summarize and/or present the charting results as they relate to the review questions and objectives. | N/A | *Protocol manuscript. Synthesis of results will be reported in the full scoping review publication, expected late 2026.* |
| **DISCUSSION** | | | | |
| Summary of evidence | **19** | Summarize the main results (including an overview of concepts, themes, and types of evidence available), link to the review questions and objectives, and consider the relevance to key groups. | **10–11** | *Discussion: Principal Findings subsection presents anticipated findings linked to each of the three review objectives, including expected heterogeneity, underrepresentation of upstream approaches, and gaps in Africentric frameworks.* |
| Limitations | **20** | Discuss the limitations of the scoping review process. | **11** | *Discussion: Strengths and Limitations subsection addresses language restriction, BCA population heterogeneity, and limits of indexed grey literature.* |
| Conclusions | **21** | Provide a general interpretation of the results with respect to the review questions and objectives, as well as potential implications and/or next steps. | **11–12** | *Discussion: Future Directions and Dissemination Plan subsections address implications and next steps. Conclusions also stated in the abstract.* |
| **FUNDING** | | | | |
| Funding | **22** | Describe sources of funding for the included sources of evidence, as well as sources of funding for the scoping review. Describe the role of the funders of the scoping review. | **12** | *Funding section: supported by a CIHR trainee award for the corresponding author's doctoral studies. Funder had no role in design, decision to publish, or manuscript preparation. Funding of included sources will be reported in the full review.* |

**Notes and Definitions**

* Where sources of evidence (see second footnote) are compiled from, such as bibliographic databases, social media platforms, and websites.

† A more inclusive/heterogeneous term used to account for the different types of evidence or data sources (e.g., quantitative and/or qualitative research, expert opinion, and policy documents) that may be eligible in a scoping review as opposed to only studies.

‡ The frameworks by Arksey and O’Malley and Levac and colleagues and the JBI guidance refer to the process of data extraction in a scoping review as data charting.

§ The process of systematically examining research evidence to assess its validity, results, and relevance before using it to inform a conclusion. Risk of bias assessment is optional in scoping reviews.

From: Tricco AC, Lillie E, Zarin W, O’Brien KK, Colquhoun H, Levac D, et al. PRISMA Extension for Scoping Reviews (PRISMA-ScR): Checklist and Explanation. Ann Intern Med. 2018;169:467–473. doi:10.7326/M18-0850.
